# Robbing the Hospitals: The Homœopathic Hospital Case

**Published:** 1916-01-29

**Authors:** R. H. Caird

**Affiliations:** Chairman. London Homœopathic Hospital. 4 Queen's Gate Place, S.W.


*To the Editor of* The Hospital.

### Robbing the Hospitals: The Homœopathic Hospital Case.

*To the Editor of* The Hospital.

Sir,—I entirely agree with what Lord Knutsford says in his letter published in The Hospital, and in the hope that other hospitals may take warning from our unfortunate experience I give you the following particulars of the fraud.

The thief stole such amounts of the cash as would exactly tally with the amount of a few cheques at the close of the year, and held those cheques back till after the bankers had given their certificate of the balance at December 31. The certificate therefore agreed with the balance required by the hospital books. He then issued the cheques, and in due course they appeared in the pass-book as paid in January. He then, before the audit, erased these cheques in January and inserted them in the pass-book as paid in December.

The auditors ticked them off in the pass-book but did not check the casts in the pass-book; had they done so the fraud would have been discovered at the first audit after it was committed.

When the cash and cheques were given to him to pay in he exhibited the pay-in slip and counterfoil properly made out, but afterwards made out another pay-in slip, omitting the cash he meant to steal. The remedy is never to let the clerk who keeps the books and must have access to the pass-book have any handling of the cash. This, of course, I have now arranged, but this particular clerk had been in the service for eleven years and was entirely trusted.

If the auditors made a practice of checking the pass-book casts such a fraud would be discovered without fail.

Pearce was tried at Bow Street last Friday, pleaded guilty, and was sentenced to eight months' imprisonment.—Yours faithfully, R. H. Caird, Chairman.

London Homœopathic Hospital.

4 Queen's Gate Place, S.W., January 24, 1916.

